# Nontuberculous Mycobacterium Peritonitis in Patients on Peritoneal Dialysis: A Scoping Review

**DOI:** 10.3390/microorganisms14030550

**Published:** 2026-02-27

**Authors:** Hiroshi Tamura, Keishiro Furuie, Hiroko Nagata, Hitoshi Nakazato, Shohei Kuraoka

**Affiliations:** Department of Pediatrics, Faculty of Life Sciences, Kumamoto University, 1-1-1 Honjo, Chuo-ku, Kumamoto 860-8556, Japan; furuie.keishiro@kuh.kumamoto-u.ac.jp (K.F.); nagata.hiroko@kuh.kumamoto-u.ac.jp (H.N.); hnakazato@kuh.kumamoto-u.ac.jp (H.N.); skuraoka@kuh.kumamoto-u.ac.jp (S.K.)

**Keywords:** nontuberculous mycobacteria, peritoneal dialysis, peritonitis, treatment

## Abstract

Early and accurate identification of causative microorganisms is essential for improving outcomes in peritoneal dialysis (PD)-associated peritonitis. However, nontuberculous mycobacterial (NTM) peritonitis remains difficult to diagnose and manage, often resulting in delayed treatment and unfavorable clinical outcomes. We conducted a scoping review to summarize the clinical features, microbiological profiles, treatment strategies, and outcomes of PD-associated NTM peritonitis. A total of 107 patients from 81 published reports were identified, including one patient treated at our institution. The mean age was 50.1 years, with a slight male predominance. Diabetes mellitus was the most common underlying cause of end-stage renal disease. Abdominal pain, fever, and cloudy dialysate were the most frequently reported symptoms, and exit-site infection was present in 55% of cases. Rapid-growing NTM species predominated, with *Mycobacterium fortuitum* being the most frequently identified organism. A substantial delay was observed between symptom onset and initiation of appropriate therapy. The mean duration of antimicrobial treatment was six months. PD catheters were removed in 90% of patients, and 69% were permanently transitioned to hemodialysis. The overall mortality rate during treatment was 18%. These findings suggest that NTM infection should be considered in cases of culture-negative peritonitis unresponsive to standard antibiotics. Early catheter removal combined with prolonged multidrug antimicrobial therapy for at least six months may be beneficial. In pediatric patients, temporary conversion to hemodialysis followed by PD catheter reinsertion or renal transplantation may represent a reasonable management option after successful infection control.

## 1. Introduction

Peritoneal dialysis (PD)-associated infections, including catheter-related infections and peritonitis, are major complications that can lead to catheter removal, conversion to hemodialysis, and increased mortality [1,2]. Prompt identification of causative microorganisms and early initiation of appropriate antimicrobial therapy are critical for preventing treatment failure and improving clinical outcomes [1,3]. The most common pathogens responsible for PD-associated peritonitis are coagulase-negative staphylococci and *Staphylococcus aureus*, which are generally identifiable using routine culture methods and respond well to standard antimicrobial regimens [4,5].

In contrast, less common pathogens such as fungi, *Mycobacterium tuberculosis*, and nontuberculous mycobacteria (NTM) are frequently associated with culture-negative peritonitis and unfavorable outcomes [6,7,8,9]. NTMs are ubiquitous environmental organisms commonly found in soil and water [10]. Their isolation is often difficult in non-specialized laboratories, and routine bacterial cultures frequently fail to detect these organisms, leading to delayed diagnosis. Although PD-associated NTM peritonitis is relatively rare, it represents a clinically important complication with limited standardized diagnostic and therapeutic strategies, and evidence-based guidelines remain insufficient [11]. Given the rarity of this condition and the heterogeneity of published reports, a comprehensive synthesis of available evidence is needed to improve awareness and guide clinical management. Because the existing literature consists predominantly of heterogeneous case reports and small case series with variable diagnostic methods, treatment approaches, and outcome reporting, quantitative synthesis or meta-analysis is not feasible. Therefore, a scoping review was considered the most appropriate design to comprehensively map the current evidence and summarize clinical patterns and management practices. Accordingly, we conducted a scoping review of published case reports and case series of PD-associated NTM peritonitis. The objectives were to characterize patient demographics, clinical manifestations, microbiological features, treatment strategies, and outcomes, and to describe practical management considerations based on the accumulated evidence.

## 2. Materials and Methods

### 2.1. Search Strategy and Study Selection

This scoping review was conducted in accordance with the Scoping Reviews (PRISMA-ScR), and reporting elements were additionally aligned with PRISMA 2020 where applicable [12]. The review protocol was retrospectively registered in the Open Science Framework (OSF) and is publicly available at: https://doi.org/10.17605/OSF.IO/GURKP (accessed on 23 February 2026). A systematic literature search was performed using PubMed, Google Scholar, and Ichushi (a major Japanese medical database), including articles published up to 31 December 2024. The search terms included “dialysis”, “peritoneal dialysis”, and “nontuberculous mycobacteria”.

Studies were excluded if they did not involve PD-associated NTM peritonitis, described NTM peritonitis in non-PD patients, involved *Mycobacterium tuberculosis*, or were conference abstracts. Articles published in languages other than English or Japanese were also excluded.

After initial screening of titles and abstracts, full texts of eligible articles were reviewed. Reference lists of included studies were manually screened to identify additional relevant publications. Only case reports and case series with microbiologically confirmed NTM peritonitis based on positive peritoneal fluid culture were included.

PD catheter removal was defined as removal of the catheter during an active episode of NTM peritonitis. NTM-related mortality was defined as death occurring during the treatment period, regardless of attribution.

For each case, data were extracted on age, sex, cause of end-stage renal disease (ESRD), clinical presentation, time from symptom onset to initiation of anti-NTM therapy, microbiological findings, antimicrobial therapy, catheter management, and clinical outcome. Two independent reviewers screened articles and extracted data, with discrepancies resolved by consensus.

A complete list of historical case reports included in this scoping review is provided in Supplementary Materials.

### 2.2. Statistical Analysis

Statistical analyses were performed for descriptive and exploratory purposes only to summarize clinical characteristics and to identify potential trends or associations between variables. Categorical variables were summarized as frequencies and percentages. Continuous variables were reported as means with standard deviations or medians with ranges, as appropriate. Group comparisons were conducted using the Mann–Whitney U test. Exploratory logistic regression analyses were performed to examine possible associations with mortality. Because this study was designed as a scoping review of heterogeneous case reports and case series, these analyses were considered hypothesis-generating rather than confirmatory, and no causal inference was intended. Accordingly, results are interpreted descriptively. All analyses were conducted using R version 4.1.3.

## 3. Results

### 3.1. Literature Review

**Figure 1:**
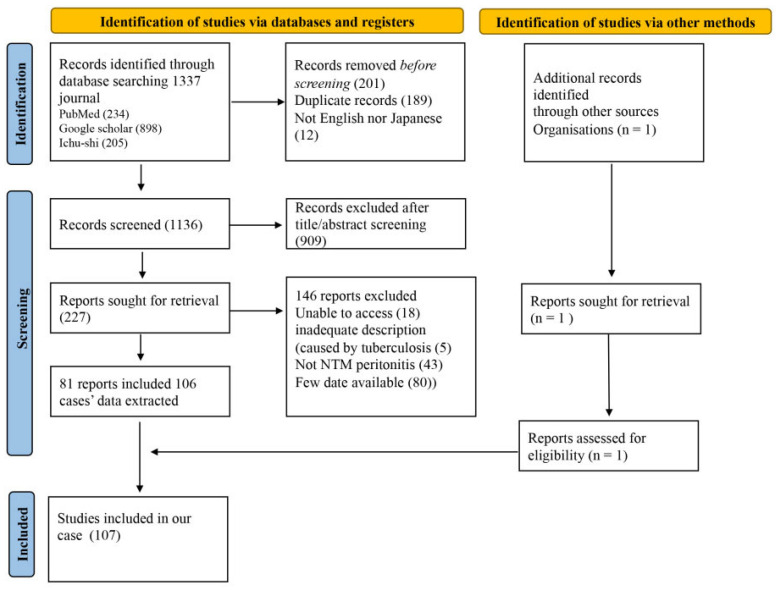
Search strategy and selection of studies.

The study selection process is shown in Figure 1. A total of 1337 articles were identified, of which 81 publications describing 106 patients met the inclusion criteria. Together with one additional institutional case, 107 patients were included in the final analysis.

### 3.2. Clinical Characteristics of Patients with NTM Peritonitis

The clinical characteristics of all patients are summarized in Table 1. The mean age was 50.1 years, and 61% of patients were male. Diabetes mellitus (40%) and glomerulonephritis (19%) were the most common causes of ESRD. None of the reports described prior immunosuppressive therapy.

The mean duration from initiation of PD to onset of NTM peritonitis was 25.0 months. The most frequently reported symptoms were abdominal pain (69%), fever (64%), and cloudy dialysate (55%). Exit-site infection (ESI) was present in 55% of cases.

Rapid-growing NTM species were the predominant pathogens. *Mycobacterium fortuitum* (33%) and *M. abscessus* (28%) were the most frequently identified organisms (Supplementary Table S1).

The mean interval from symptom onset to initiation of appropriate therapy was 16.4 days (median, 12 days). The mean therapy duration was six months (median, four months). PD catheters were removed in 90% of cases, and 69% of patients were permanently transitioned to hemodialysis. Overall mortality during therapy was 18%.

### 3.3. Comparison Between Rapid-Growing and Slow-Growing NTM Peritonitis

Patients with slow-growing NTM peritonitis experienced a significantly longer delay between symptom onset and initiation of therapy compared with those infected with rapid-growing NTM. Clinical outcomes were also significantly poorer in the slow-growing NTM group (Table 2).

For slow-growing NTM infections, rifampin, ethambutol, isoniazid, and macrolides were most frequently used. In contrast, treatment of rapid-growing NTM peritonitis commonly included amikacin, macrolides, and quinolones (Supplementary Table S2).

### 3.4. Comparison Between Adult and Pediatric Patients

Comparison between adult and pediatric patients revealed a significant difference in the underlying cause of ESRD. Diabetes mellitus was the predominant etiology among adults, whereas congenital anomalies of the kidney and urinary tract were most common in pediatric patients.

Recovery rates were 80% in adults and 92% in pediatric patients. Although fewer pediatric patients required permanent transition to hemodialysis, this difference was not statistically significant (Table 3).

### 3.5. Multivariate Analysis of Treatment Success and Mortality

Multivariate logistic regression analysis identified slow-growing NTM species as the only factor significantly associated with increased mortality (odds ratio 5.48; 95% confidence interval 1.36–22.7; *p* = 0.03) (Table 4).

## 4. Discussion

This scoping review provides a comprehensive overview of PD-associated NTM peritonitis, highlighting its diagnostic challenges, complex management, and substantial mortality. Although relatively rare, NTM peritonitis appears to be a clinically important complication of PD, frequently resulting in delayed diagnosis, prolonged antimicrobial therapy, PD catheter removal, and permanent transition to hemodialysis.

Impaired cell-mediated immunity in patients with end-stage renal disease (ESRD) likely contributes to susceptibility to NTM infection [13]. Declining kidney function is associated with reduced B-lymphocyte and CD4-positive T-lymphocyte counts, as well as impaired T-cell and neutrophil function, leading to compromised cellular and humoral immunity. Additional factors, including uremic toxins, oxidative stress, vascular endothelial dysfunction, chronic inflammation, and abnormalities in bone and mineral metabolism, may further weaken host defenses and increase infection risk [14].

Although the precise immune mechanisms protecting the peritoneal cavity are not fully understood, several studies suggest that PD itself may impair phagocytic and lymphocytic activity within peritoneal fluid, thereby increasing susceptibility to infection even with relatively low bacterial loads [13,15]. While some patients with NTM peritonitis had underlying immune-related conditions such as systemic lupus erythematosus or glomerulonephritis, the use of corticosteroids or immunosuppressive agents was not consistently reported. Nevertheless, such therapies are known risk factors for NTM infection. Diabetes mellitus, a condition associated with impaired antibacterial immunity, was the most common cause of ESRD in this cohort, suggesting its potential role as a risk factor. In addition, individuals with human immunodeficiency virus (HIV) infection are at increased risk for NTM disease [16], and two patients in this review were living with HIV. Collectively, these findings suggest that most PD patients who develop NTM peritonitis are immunocompromised to some degree.

Consistent with previous reports [7,17], rapid-growing NTM species—including *Mycobacterium fortuitum*, *M. abscessus*, and *M. chelonae*—were the predominant causative organisms in PD-associated NTM peritonitis. In contrast, slow-growing species such as *M. avium*, which are the primary pathogens in pulmonary NTM disease [18,19], were relatively uncommon, particularly in cases involving ESI. This difference may reflect variations in environmental niches and routes of exposure among NTM species.

A substantial diagnostic delay was observed, with an average of approximately 17 days between symptom onset and initiation of appropriate therapy. Early diagnosis of NTM peritonitis is particularly challenging because its clinical presentation closely resembles that of bacterial or tuberculous peritonitis [20,21]. Common symptoms—including fever, abdominal pain, and cloudy dialysate—are nonspecific and frequently overlap with other causes of PD-associated peritonitis. Additional symptoms such as anorexia, fatigue, nausea, vomiting, and weight loss have also been reported [22,23]. Laboratory findings, including leukocyte counts and differentials in both peritoneal effluent and peripheral blood, are variable and do not reliably distinguish NTM peritonitis from infections caused by *Mycobacterium tuberculosis* or conventional bacteria [20,21].

Notably, many patients with NTM peritonitis had concurrent ESI in which organisms other than NTM were initially identified. This may be attributable to routine culture practices, in which exit-site swabs are typically processed using standard bacterial culture methods, while mycobacterial cultures are often reserved for cases with negative results. Common skin flora such as *Staphylococcus aureus* and coagulase-negative staphylococci grow more rapidly than NTM species, potentially leading to underdiagnosis of NTM in ESI [24,25]. Furthermore, NTM can colonize medical equipment and solutions [25,26,27], and although evidence is limited, person-to-person transmission has been suggested in certain settings [28,29]. NTM peritonitis may also occur following medical procedures; in one of our cases, infection developed after PD catheter replacement [30].

NTM species exhibit intrinsic resistance to many first-line empirical antibiotics commonly used for PD-associated infections. Prior studies have suggested that gentamicin use at the exit site may increase the risk of NTM-related ESI [31]. Although disinfectants such as chlorhexidine and hydrogen peroxide are commonly recommended for exit-site care, some NTM species demonstrate resistance to chlorhexidine. In addition, antiseptics such as povidone–iodine and hydrogen peroxide may cause local skin damage, potentially increasing susceptibility to infection [32,33].

From a microbiological perspective, rapid-growing NTM typically become detectable within 3–5 days of culture, whereas slow-growing species may require at least 7 days or longer. Acid-fast bacillus (AFB) staining of peritoneal effluent can facilitate earlier detection; however, AFB smears may be negative in NTM infection and cannot differentiate NTM from *M. tuberculosis*. Because routine bacterial cultures are often discarded after 4–5 days, clinicians should notify microbiology laboratories when NTM infection is suspected to ensure prolonged incubation and the use of selective mycobacterial media, such as Löwenstein–Jensen medium [34].

Accurate identification of the causative NTM species appears to be important, as antimicrobial susceptibility patterns vary considerably among species. Although the Runyon classification system remains useful for initial categorization based on growth rate and pigment production [26,27], molecular diagnostic methods—including 16S rRNA sequencing and commercially available probes—are increasingly important for rapid and accurate identification. Rapid-growing NTM may be misidentified as Gram-positive rods, such as diphtheroids or *Corynebacterium* species, which can delay appropriate diagnosis and therapy [35,36]. This issue has been highlighted in both quality control studies and international PD guidelines [11,34].

The mean treatment duration in this cohort was six months, which is shorter than that recommended for pulmonary NTM disease [27]. Treatment regimens varied widely and were tailored according to species identification and susceptibility testing. Rapid-growing NTM were commonly treated with combinations including aminoglycosides, macrolides, quinolones, carbapenems, and tetracyclines, whereas slow-growing species were often treated with rifampin, ethambutol, isoniazid, and macrolides. Given the lack of standardized regimens and the limited evidence from controlled studies, combination therapy guided by antimicrobial susceptibility testing and clinical response may be appropriate.

Based on our institutional experience, we suggest combination therapy with at least two to three antimicrobial agents, continued for a minimum of six months. Antibiotic selection should consider drug susceptibility, patient age, potential adverse effects, and local resistance patterns. Consultation with specialists experienced in managing NTM infections is particularly important, especially in pediatric patients.

Our analysis of recent case series with PD-associated NTM infections showed that the PD catheters were eventually removed in most patients with NTM peritonitis (95/105, 90%), PD continuation was possible in approximately 21% of the recovered patients (Table 1). However, among the 10 patients whose PD catheter was not removed, 5 died from causes related to NTM peritonitis. In most cases, the PD catheter was removed or replaced, after which the infection resolved, suggesting that catheter removal may play an important role in infection control.

Among the pediatric patients who switched to another modality after hemodialysis, 30% transitioned to PD and 50% renal transplantation (RT). The mean duration of hemodialysis was 2 months for patients who underwent PD and 14 months for those who underwent RT.

In most adult cases the PD catheter was reinserted after 6 months, while in pediatric cases, catheter reinsertion occurred on average 8 weeks after removal.

Complete pathogen eradication before transplantation is critical because NTM infections can recur after a median of 37 months (range 3 days–252 months) following kidney transplantation, indicating the reactivation of latent infection [37].

Immunosuppressive drugs after RT are a potential risk factor for recurrent NTM infection, which can be fatal and can cause rejection because dosages of immunosuppressive drugs are lowered during treatment for NTM infection [38,39].

Although evidence regarding the optimal interval between completion of antimycobacterial therapy and RT remains limited, patients awaiting transplantation may be monitored for at least six months to confirm sustained microbiological cure.

Future research involving large patient cohorts is essential to determine the most effective treatment regimens, appropriate durations as well as the appropriate timing for RT after treatment for NTM peritonitis.

In pediatric patients, temporary conversion to hemodialysis followed by PD catheter reinsertion or kidney transplantation was feasible after infection control. Complete eradication of NTM before transplantation is critical, as recurrence has been reported months to years after RT [37]. Immunosuppressive therapy following transplantation may further increase the risk of recurrent or disseminated NTM infection [38,39]. Although optimal timing remains uncertain, careful monitoring for at least six months after completion of antimycobacterial therapy may be reasonable before proceeding with transplantation.

**Figure 2:**
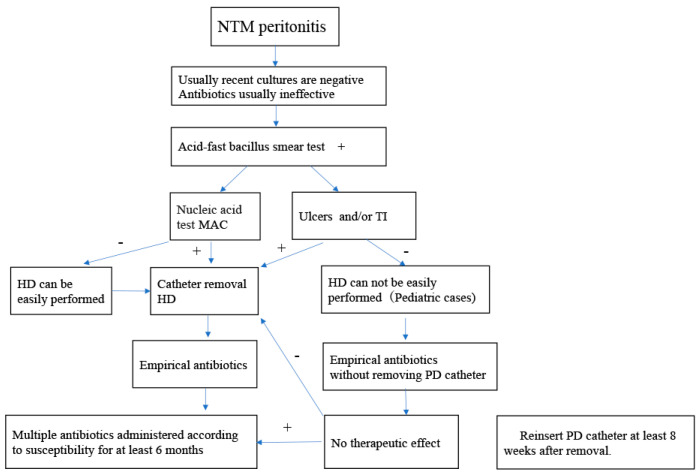
Proposed management algorithm for peritoneal dialysis-associated nontuberculous mycobacterial (NTM) peritonitis.

Based on the available evidence and our clinical experience, we propose a management algorithm for PD-associated NTM peritonitis (Figure 2).

NTM infection should be suspected in cases of culture-negative peritonitis that are unresponsive to standard empirical antibiotics. When acid-fast bacillus staining is positive, polymerase chain reaction testing is recommended to differentiate *Mycobacterium tuberculosis* from nontuberculous mycobacteria. PD catheter removal should be considered in patients with confirmed NTM infection, particularly in the presence of ulceration, tunnel infection, or involvement of the deep cuff. Empirical multidrug antimicrobial therapy targeting NTM should be initiated and subsequently adjusted according to antimicrobial susceptibility testing. Prolonged combination therapy for at least six months is generally required. Temporary conversion to hemodialysis may be necessary, with consideration of PD catheter reinsertion after adequate infection control (approximately 8 weeks in pediatric patients and 6 months in adults). Renal transplantation should be considered only after sustained microbiological cure.

Although rare, NTM peritonitis was associated with a high mortality rate (18%) in our review, consistent with previously reported mortality rates of 14.3% and 17.2% despite antimicrobial therapy [7,40]. In our multivariate analysis, the causative organism was identified as the only significant risk factor for mortality. However, univariate analysis comparing rapid-growing and slow-growing NTM also demonstrated a significant difference in the delay to initiation of appropriate therapy. Given the diagnostic difficulty associated with slow-growing NTM, treatment delay itself may contribute to poorer outcomes. However, because comorbid conditions were not fully accounted for, it remains unclear whether mortality was directly attributable to peritonitis, and the observed mortality rate may have been influenced by additional unmeasured confounding factors.

Notably, no deaths were reported among pediatric patients aged ≤15 years. This favorable outcome may reflect the fact that pediatric PD is typically managed in highly specialized medical centers. In contrast, adult patients who required long-term hemodialysis following PD catheter removal were often managed in general hospitals, where delayed pathogen identification and a higher burden of complications may have contributed to worse outcomes [41]. In children, hemodialysis is technically challenging because of difficulties in vascular access and the frequent need for sedation, and therefore can be provided only at a limited number of specialized centers. Consequently, when PD-associated ESI or peritonitis caused by NTM is suspected in pediatric patients, early referral to specialized centers and consultation with clinicians experienced in NTM management of NTM may be beneficial [38].

Several limitations of this study should be acknowledged. The available evidence consisted primarily of retrospective case reports and case series, with substantial heterogeneity in diagnostic approaches, antimicrobial regimens, and outcome reporting. Publication bias and incomplete clinical data limited robust assessment of causal relationships between management strategies and outcomes.

## 5. Conclusions

Nontuberculous mycobacterial peritonitis remains a rare but clinically significant complication of peritoneal dialysis, often associated with delayed diagnosis, prolonged therapy, frequent catheter removal, and considerable morbidity and mortality. Early recognition, prompt PD catheter removal, and prolonged combination antimicrobial therapy tailored to the causative species appear to be important for successful management. In pediatric patients, temporary conversion to hemodialysis followed by PD catheter reinsertion or kidney transplantation may be considered after adequate infection control. Further prospective studies are required to establish standardized diagnostic and therapeutic strategies.

## Figures and Tables

**Table 1:** Clinical characteristics of patients with PD-related NTM peritonitis.

Age (years), N = 107	Mean 50.1 ± 20.2 Median 54 (0.4~86)
Sex	Male 65/107 (61%)
Kidney disease	DM 34/84 (40%) GN 15/84 (19%) Sclerosis 8/84 (10%) CAKUT 8/84 (10%) FSGS 4/84 (5%) Lupus 4/84 (5%) HT 3/84 (4%) Others 8/84 (10%) (HIV 2, Drugs 2, ARF 1, CNS1, HUS 1, Nephrolithiasis 1)
Dialysis duration before onset of NTM (months), N = 82	Mean 25.0 ± 23.4 Median 18 (0.3~96)
Clinical manifestations	Abdominal pain 66/95 (69%) Fever 61/95 (64%) Cloudy fluid 52/95 (55%) Nausea, vomiting 11/95 (12%)
RGM/(RGM + SGM)	86/106 (81%)
Duration from disease onset to anti-NTM treatment (days), N = 79	Mean 16.4 ± 12.2 Median 12 (3~50)
Catheter remove	95/105 (90%)
ESI *1	53/97 (55%)
Duration of medication (months), N = 72	Mean 6.0 ± 4.1 Median 4 (0.5~18)
Outcome	recovery 78/95 (82%) [HD 54/78 (69%) PD 16/78 (21%) (not remove 6) RT 8/78 (10%)] *2 All-cause mortality 17/95 (18%) *3

*1. includes infections other than NTM infections. *2. PD, RT includes patients who switched from HD. Six patients were able to continue on PD without having their catheters removed. *3. All-cause mortality (death from NTM peritonitis or while receiving antituberculosis treatment for NTM peritonitis).

**Table 2:** Comparison of clinical characteristics between patients with rapid-growing (RGM) and slow-growing (SGM) NTM peritonitis.

	SGM (20)	RGM (86)	*p* Value
Age (years)	Mean 46.7 ± 22.1 Median 50 (4~80)	Mean 50.9 ± 20.4 Median 55 (0.4~86)	*p* = 0.34
Sex	M 10/20 (50%)	M 55/86 (64%)	*p* = 0.14
Kidney disease	DM 2/14 (14%) GN 1/14 (7%) Sclerosis 3/14 (21%) CAKUT 1/14 (7%) FSGS 2/14 (14%) Lupus 1/14 (7%) Others 4/14	DM 31/69 (45%) GN 14/69 (20%) Sclerosis 5/69 (7%) CAKUT 6/69 (9%) FSGS 2/69 (3%) Lupus 3/69 (4%) Others 8/69	*p* < 0.01 *1
Dialysis duration before onset of NTM (months)	Mean 28.1 ± 24.3 Median 24 (0.3~60) N = 16	Mean 24.8 ± 24.3 Median 16 (0.5~96) N = 66	*p* = 0.30
Clinical manifestations	Fever 10/18 (56%) Abdominal pain 11/18 (61%) Nausea, vomiting 1/18 (6%) Cloudy fluid 10/18 (56%)	Fever 46/76 (61%) Abdominal pain 55/76 (74%) Nausea, vomiting 10/76 (13%) Cloudy fluid 42/76 (55%)	
Duration from disease onset to anti-NTM treatment (days)	Mean 26.8 ± 12.5 Median 27 (6~50) N = 16	Mean 14.0 ± 10.5 Median 10 (3~49) N = 63	*p* < 0.01
Catheter remove	17/20 (85%)	77/84 (92%)	*p* = 0.48
ESI	4/19 (21%)	48/77 (62%)	*p* < 0.01
Duration medication (months)	Mean 10.8 ± 4.6 Median 12 (4~18) N = 10	Mean 5.4 ± 3.4 Median 6 (1~15) N = 62	*p* = 0.01
Outcome	recovery 10/17 (59%) [HD 8/11 (73%) PD 1/11 (9%) (not remove 0) RT 1/11 (9%)] *2 All-cause mortality 7/17 (41%) *3	recovery 68/78 (87%) [HD 46/68 (68%) PD 15/68 (22%) (not remove 5) RT 7/68 (10%)] *2 All-cause mortality 10/78 (13%) *3	*p* < 0.01

*1. A comparison was made between DM and other diseases. *2. PD, RT includes patients who switched from HD. *3. The three patients whose PD catheters were not removed did not consent to removal and died. All-cause mortality (death from NTM peritonitis or while receiving antituberculosis treatment for NTM peritonitis).

**Table 3:** Comparison of clinical characteristics between patients with adult and pediatric NTM peritonitis.

	Adults (92)	Children (15)	*p* Value
Age (years)	Mean 56.8 ± 13.2 Median 56 (28–86)	Mean 9.14 ± 5.42 Median 9 (0.4~17)	
Sex	M 55/92 (60%)	M 10/15 (67%)	*p* = 0.62
Kidney disease	DM 34/71 (48%) GN 14/71 (20%) Sclerosis 8/71 (11%) CAKUT 4/71 (6%) FSGS 1/71 (1%) Lupus 2/71 (3%) Others 8/71	DM 0/13 (0%) GN 1/13 (11%) Sclerosis 0/13 (0%) CAKUT 4/13 (44%) FSGS 3/13 (11%) Lupus 2/13 (0%) Others 3/13 (CNS1, drugs2)	*p* < 0.01 *1
Dialysis duration before onset of NTM (months)	Mean 25.4 ± 24.1 Median 18 (0.3~96) N = 69	Mean 18.5 ± 15.88 Median 8.5 (1~48) N = 12	*p* = 0.46
Clinical manifestations	Fever 48/80 (60%) Abdominal pain 57/80 (71%) Nausea, vomiting 10/80 (13%) Cloudy fluid 40/80 (50%)	Fever 13/15 (87%) Abdominal pain 9/15 (60%) Nausea, vomiting 1/15 (7%) Cloudy fluid 11/15 (73%)	
RGM/(RGM + SGM)	55/71 (77%)	11/15 (73%)	*p* = 0.40
Duration from disease onset to anti-NTM treatment (days)	Mean 16.2 ± 11.1 Median 12.5 (4~50) N = 66	Mean 18.14 ± 15.68 Median 8.5 (3~40) N = 14	*p* = 0.63
Catheter remove	81/90 (90%)	14/15 (93%)	*p* = 0.61
ESI	44/82 (54%)	9/15 (60%)	*p* = 0.63
Duration medication (months)	Mean 5.4 ± 3.9 Median 5 (1~18) N = 64	Mean 8.3 ± 3.9 Median 8.5 (2~15) N = 12	*p* = 0.02
Outcome	recovery 66/82 (80%) [HD 51/66 (77%) PD 13/66 (20%) (not remove 5) RT 2/66 (3%)] *2 All-cause mortality 16/82 (20%) *3	recovery 12/13 (92%) [HD 3/12 (25%) PD 3/12 (25%) (not remove 1) RT 6/12 (50%)] *2 All-cause mortality 1/13 (8%) *3	*p* = 0.32

*1. A comparison was made between DM and other diseases. *2. PD, RT includes patients who switched from HD. *3. The three patients whose PD catheters were not removed did not consent to removal and died. All-cause mortality (death from NTM peritonitis or while receiving antituberculosis treatment for NTM peritonitis).

**Table 4:** Multivariate logistic regression analysis of factors associated with mortality.

	*p* Value	OR	95%CI (Low)	95%CI (High)
Age	0.24	0.35	0.01	2.72
Sex	0.79	1.79	0.49	6.97
Kidney disease *1	0.80	0.63	0.16	2.62
Dialysis duration before onset of NTM	0.27	0.74	0.18	2.97
Duration from disease onset to anti-NTM treatment	0.42	0.86	0.19	3.84
Catheter removal	0.16	5.13	0.89	28.8
Slow-growing NTM species	0.03	5.48	1.36	22.7

*1. A comparison was made between DM and other diseases.

## Data Availability

No new data were created or analyzed in this study. Data sharing is not applicable to this article.

## Supplementary Materials

The following supporting information can be downloaded at https://www.mdpi.com/article/10.3390/microorganisms14030550/s1, Table S1: Classification of causative nontuberculous mycobacterial species in 107 cases of PD-associated peritonitis; Table S2: Literature Review of Treatment of NTM peritonitis. Refs. [17,22,23,40,42,43,44,45,46,47,48,49,50,51,52,53,54,55,56,57,58,59,60,61,62,63,64,65,66,67,68,69,70,71,72,73,74,75,76,77,78,79,80,81,82,83,84,85,86,87,88,89,90,91,92,93,94,95,96,97,98,99,100,101,102,103,104,105,106,107,108,109,110,111,112,113,114,115,116,117,118] are included in the Supplementary Materials.

## Abbreviations

PD	peritoneal dialysis
APD	automated peritoneal dialysis
HD	Hemodialysis
CHD	continuous hemodialysis
RT	renal transplantation
NTM	nontuberculous *Mycobacteria*
SGM	slow-growing nontuberculous *Mycobacteria*
RGM	rapid-growing nontuberculous *Mycobacteria*
FSGS	focal segmental glomerulosclerosis
GN	Glomerulonephritis
DM	diabetes mellitus
CAKUT	Congenital anomalies of the kidney and urinary tract
HT	Hypertension
HIV	Human Immunodeficiency Virus
ARF	acute renal failure
CNS	Congenital nephrotic syndrome
HUS	Hemolytic uremic syndrome
ESI	exit-site infection
TI	tunnel infection
NFLX	Norfloxacin
AMK	Amikacin
IPM/CS	imipenem/cilastatin sodium
CPFX	ciprofloxacin hydrochloride
CAM	Clarithromycin
REP	Rifampin
EB	Ethambutol
INH	Isoniazid
RBT	Rifabutin
PZA	Pyrazinamide
TOB	Tobramycin
AZM	Azithromycin Hydrate
EM	Erythromycin
MFLX	Moxifloxacin Hydrochloride
LVFX	Levofloxacin Hydrate
MEPM	Meropenem Hydrate
DOXY	Doxycycline
MINO	Minocycline Hydrochloride
TC	Tetracycline
