# Supplementary material for: Nontuberculous Mycobacterium Peritonitis in Patients on Peritoneal Dialysis: A Scoping Review
Source: Microorganisms. 2026 Feb 27;14(3):550. doi: 10.3390/microorganisms14030550 (PMC13029714; doi:10.3390/microorganisms14030550)
Supplement: Supplementary file 1 [file microorganisms-14-00550-s001.zip › NTM table S1.pdf]

Table S1. **Classification of causative nontuberculous mycobacterial species in 107 cases of PD-associated peritonitis**

		Peritonitis N=107
SGM Type1	*Mycobacterium Kansasii*	2
	Mycobacterium Simiae	1
SGM Type2	*Mycobacterium Gordonae*	2
	Mycobacterium Heckeshornense	1
	Type2 Mycobacterium	2
SGM Type 3	*MAC*	10
	Mycobacterium Xenpi	1
	Mycobacterium Gastri	1
RGM Type 4	*Mycobacterium Fortuitum*	35
	Mycobacterium Abscessus	30
	Mycobacterium Chelonae	10
	Mycobacterium Smegmatis	2
	Mycobacterium Wolinskyi	2
	Mycobacterium Phlei	1
	Mycobacterium Rhodesiae	1
	Mycobacterium Porcinum	1
	Mycobacterium Iranicum	1
	Mycobacterium Septicum	1
	Mycobacterium Massiliense	1
	Mycobacterium Chlorophenolicum	1
unknown	*Mycobacterium Paraoxydans*	1
