# Supplementary material for: Nontuberculous Mycobacterium Peritonitis in Patients on Peritoneal Dialysis: A Scoping Review
Source: Microorganisms. 2026 Feb 27;14(3):550. doi: 10.3390/microorganisms14030550 (PMC13029714; doi:10.3390/microorganisms14030550)
Supplement: Supplementary file 1 [file microorganisms-14-00550-s001.zip › NTM table S2.pdf]

Table S2. **Literature Review of Treatment of NTM peritonitis.**

	Aminoglycosides		Macrolides			New quinolones			Carbapenems		Tetracyclines		
	AMK	TOB	CAM	AZM	EM	CPFX	MFLX	LVFX	IPM/CS	MEPM	DOXY	MINO	TC
Mycobacterium fortuitum N=35	17	1	11	0	1	9	1	4	2	0	6	1	1
Mycobacterium abscessus N=30	25	0	23	2	0	4	2	0	7	1	0	0	0
Mycobacterium chelonae N=11	6	2	5	1	0	1	1	1	0	0	1	0	0

	Antituberculous drugs					Aminoglycosides	Macrolides		New quinolones	
	REP	EB	INH	RBT	PZA	AMK	CAM	AZM	CPFX	LVFX
MAC N=10	6	7	3	1	1	3	4	1	1	1
Mycobacterium kansasii N=2	2	1	2	1	0	0	0	0	0	0
