# Supplementary material for: Nontuberculous Mycobacterium Peritonitis in Patients on Peritoneal Dialysis: A Scoping Review
Source: Microorganisms. 2026 Feb 27;14(3):550. doi: 10.3390/microorganisms14030550 (PMC13029714; doi:10.3390/microorganisms14030550)
Supplement: Supplementary file 1 [file microorganisms-14-00550-s001.zip › Supplementary_References_S1.pdf]

Supplementary References S1

A comprehensive list of historical case reports and case series of peritoneal dialysis–associated nontuberculous mycobacterial peritonitis.

Pulliam, J.P.; Vernon, D.D.; Alexander, S.R.; Hartstein, A.I.; Golper, T.A. *Nontuberculous mycobacterial* peritonitis associated with continuous ambulatory peritoneal dialysis. *Am. J. Kidney Dis.* **1983**, *2*, 610–614. https://doi.org/10.1016/s0272-6386(83)80040-7.

Linton, I.M.; Leahy, S.I.; Thomas, G.W. *Mycobacterium gastri* peritonitis in a patient undergoing continuous ambulatory peritoneal dialysis. *Aust. New Zealand J. Med.* **1986**, *16*, 224–225.

LaRocco, M.T.; Mortensen, J.E.; Robinson, A. *Mycobacterium fortuitum* peritonitis in a patient undergoing chronic peritoneal dialysis. *Diagn. Microbiol. Infect Dis.* **1986**, *4*, 161–164.

Woods, G.L.; Hall, G.S.; Schreiber, M.J. *Mycobacterium fortuitum* Peritonitis Associated with Continuous Ambulatory Peritoneal Dialysis. *J. Clin. Microbiol.* **1986**, *23*, 786–788. https://doi.org/10.1128/jcm.23.4.786-788.1986.

Merlin, T.L.; Tzamaloukas, A.H. *Mycobacterium chelonae* peritonitis associated with continuous ambulatory peritoneal dialysis. *Am. J. Clin. Pathol.* **1989**, *91*, 718–720.

Soriano, F.; Rodriguez-Tudela, J.L.; Gómez-Garcés, J.L.; Velo, M. Two Possibly Related Cases of *Mycobacterium fortuitum* Peritonitis Associated with Continuous Ambulatory Peritoneal Dialysis. *Eur. J. Clin. Microbiol. Infect. Dis.* **1989**, *8*, 895–897. https://doi.org/10.1007/bf01963778.

Sennesael, J.J.; Maes, V.A.; Pierard, D.; Debeukelaer, S.H.; Verbeelen, D.L. Streptomycin pharmacokinetics in relapsing *Mycobacterium xenopi* peritonitis. *Am. J. Nephrol.* **1990**, *10*, 422–425. https://doi.org/10.1159/000168161.

Dunmire, R.B.; Breyer, J.A. Nontuberculous mycobacterial peritonitis during continuous ambulatory peritoneal dialysis: Case report and review of diagnostic and therapeutic strategies. *Am. J. Kidney Dis.* **1991**, *18*, 126–130. https://doi.org/10.1016/s0272-6386(12)80303-9.

Giladi, M.; Lee, B.E.; Berlin, O.G.W.; Panosian, C.B. Peritonitis caused by *Mycobacterium kansasii* in a patient undergoing continuous ambulatory peritoneal dialysis. *Am. J. Kidney Dis.* **1992**, *19*, 597–599. https://doi.org/10.1016/s0272-6386(12)80841-9.

Kolmos, H.J.; Brahm, M.; Bruun, B. Peritonitis with *Mycobacterium fortuitum* in a patient on continuous ambulatory peritoneal dialysis. *Scand. J. Infect. Dis.* **1992**, *24*, 801–803. https://doi.org/10.3109/00365549209062468.

Perlino, C.A. *Mycobacterium avium* complex: An unusual cause of peritonitis in patients undergoing continuous ambulatory peritoneal dialysis. *Clin. Infect. Dis.* **1993**, *17*, 1083–1084. https://doi.org/10.1093/clinids/17.6.1083.

White, R.; Abreo, K.; Flanagan, R.; Gadallah, M.; Krane, K.; Ei-Shahawy, M.; Shakamuri, S.; McCoy, R. Nontuberculous Mycobacterial Infections in Continuous Ambulatory Peritoneal Dialysis Patients. *Am. J. Kidney Dis.* **1993**, *22*, 581–587. https://doi.org/10.1016/s0272-6386(12)80932-2.

Perazella, M.; Eisen, T.; Brown, E. Peritonitis associated with disseminated *Mycobacterium avium* complex in an acquired immunodeficiency syndrome patient on chronic ambulatory peritoneal dialysis. *Am. J. Kidney Dis.* **1993**, *21*, 319–321. https://doi.org/10.1016/s0272-6386(12)80753-0.

Choi, C.W.; Cha, D.R.; Kwon, Y.J.; Cho, W.Y.; Pyo, H.J.; Kim, H.K. *Mycobacterium fortuitum* peritonitis associated with continuous ambulatory peritoneal dialysis. *Korean J. Intern. Med.* **1993**, *8*, 25–28. https://doi.org/10.3904/kjim.1993.8.1.25.

Gehr, T.W.; Walters, B.A. Catheter-Related *Mycobacterium chelonei* infection in a CAPD patient. *Perit. Dial. Int. J. Int. Soc. Perit. Dial.* **1994**, *14*, 278–279. https://doi.org/10.1177/089686089401400315.

Irizarry, L.; Rupp, J.; Smart, J.; Nwosu, A.; Tzamaloukas, A. Diagnostic difficulties with mycobacteria peritonitis in a CAPD patient. *Perit. Dial. Int. J. Int. Soc. Perit. Dial.* **1996**, *16*, 427. https://doi.org/10.1177/089686089601600421.

Harro, C.; Braden, G.L.; Morris, A.B.; Lipkowitz, G.S.; Madden, R.L. Failure to cure *Mycobacterium gordonae* peritonitis associated with continuous ambulatory peritoneal dialysis. *Clin. Infect. Dis.* **1997**, *24*, 955–957. https://doi.org/10.1093/clinids/24.5.955.

Paul, E.; Devarajan, P. *Mycobacterium phlei* peritonitis: A rare complication of chronic peritoneal dialysis. *Pediatr. Nephrol.* **1998**, *12*, 67–68. https://doi.org/10.1007/s004670050407.

Slagle, K.M.; Oblack, D.L. *Mycobacterium abscessus* peritonitis: A case report. *Clin. Lab. Sci.* **1998**, *11*, 206–208.

Vera, G.; Lew, S.Q. *Mycobacterium fortuitum* peritonitis in two patients receiving continuous ambulatory peritoneal dialysis. *Am. J. Nephrol.* **1999**, *19*, 586–589. https://doi.org/10.1159/000013524.

Kawamoto, S.; Otani, K.; Kawaguchi, Y.; Hosoya, T. *Mycobacterium Fortuitum* peritonitis associated with CAPD: Diagnosis by a molecular biology technique. *Perit. Dial. Int. J. Int. Soc. Perit. Dial.* **1999**, *19*, 592–593. https://doi.org/10.1177/089686089901900618.

Osada, S.; Hamada, C.; Fukui, M.; Kobata, M.; Shirato, I.; Tomino, Y. A case of *Mycobacterium fortuitum* peritonitis associated with continuous ambulatory peritoneal dialysis (CAPD). *Nihon Toseki Igakkai Zasshi* **2001**, *34*, 1251–1255. https://doi.org/10.4009/jsdt.34.1251.

Youmbissi, J.T.; Malik, Q.T.; Ajit, S.K.; Al Khursany, I.A.; Rafi, A.; Karkar, A. Non *Tuberculous mycobacterium peritonitis* in continuous ambulatory peritoneal dialysis. *J. Nephrol.* **2001**, *14*, 132–135.

Tang, S.; Tang, A.; Lam, W.; Cheng, Y.; Ho, Y.W. Successful Treatment of *Mycobacterium Fortuitum* peritonitis without tenckhoff catheter removal in CAPD. *Perit. Dial. Int. J. Int. Soc. Perit. Dial.* **2003**, *23*, 304–305. https://doi.org/10.1177/089686080302300318.

Ando, T.; Ryuzaki, M.; Handa, M.; Sekita, T.; Sakurai, T. *Mycobacterium fortuitum* Peritonitis in a Patient Undergoing Continuous Ambulatory Peritoneal Dialysis. *Hong Kong J. Nephrol.* **2003**, *5*, 101–104. https://doi.org/10.1016/s1561-5413(09)60116-2.

Ferrara, E.; Lemire, J.; Grimm, P.C.; Reznik, V.M.; Mendoza, S.A.; Leake, J.A.; Benador, N.M. *Mycobacterial peritonitis* in pediatric peritoneal dialysis patients. *Pediatr. Nephrol.* **2004**, *19*, 114–117. https://doi.org/10.1007/s00467-003-1348-1.

Keenan, N.; Jeyaratnam, D.; Sheerin, N.S. *Mycobacterium simiae*: A previously undescribed pathogen in peritoneal dialysis peritonitis. *Am. J. Kidney Dis.* **2005**, *45*, e75–e78. https://doi.org/10.1053/j.ajkd.2005.01.040.

Kameyama, H.; Mori, Y.; Kimura, T.; Sugishita, C.; Adachi, T.; Sonomura, K.; Kusaba, T.; Tanda, S.; Kishimoto, N.; Okigaki, M.; et al. A Case Report of *Mycobacterium abscessus* Peritonitis in a Peritoneal Dialysis Patient. *Ther. Apher. Dial.* **2007**, *11*, 449–451. https://doi.org/10.1111/j.1744-9987.2007.00526.x.

Rho, M.; Bia, F.; Brewster, U.C. *Nontuberculous mycobacterial* peritonitis in peritoneal dialysis patients. *Semin. Dial.* **2007**, *20*, 271–276. https://doi.org/10.1111/j.1525-139x.2007.00289.x.

Hod, T.; Kushnir, R.; Paitan, Y.; Korzets, Z. *Mycobacterium fortuitum* infection in continuous ambulatory peritoneal dialysis. *Clin. Nephrol.* **2008**, *70*, 546–553. https://doi.org/10.5414/cnp70546.

Lee, K.-F.; Chen, H.-H.; Wu, C.-J. *Mycobacterium fortuitum* infection in continuous ambulatory peritoneal dialysis. *Ren. Fail.* **2008**, *30*, 335–338. https://doi.org/10.1080/08860220701861185.

Curry, E.; Yehia, M.; Roberts, S. CAPD Peritonitis caused by *Mycobacterium rhodesiae*. *Perit. Dial. Int. J. Int. Soc. Perit. Dial.* **2008**, *28*, 97–99. https://doi.org/10.1177/089686080802800117.

Falcone, E.; Alam, A.; Tangri, N. *Mycobacterium avium* complex-associated peritonitis in a patient on continuous ambulatory peritoneal dialysis.. *Clin. Nephrol.* **2008**, *69*, 387–391. https://doi.org/10.5414/cnp69387.

Maeda, Y.; Uno, T.; Yoshida, A.; Takahashi, A.; Inaba, N.; Shiigai, T. *Nontuberculous mycobacterial* Peritonitis in a Patient Undergoing Continuous Ambulatory Peritoneal Dialysis. *J. Rural. Med.* **2009**, *4*, 75–79. https://doi.org/10.2185/jrm.4.75.

Chung, J.-W.; Cha, Y.-J.; Oh, D.-J.; Nam, W.-J.; Kim, S.-H.; Lee, M.-K.; Kim, H.-R. Disseminated *Mycobacterium avium* complex infection in a Non-HIV-infected patient undergoing continuous ambulatory peritoneal dialysis. *Ann. Lab. Med.* **2010**, *30*, 166–170. https://doi.org/10.3343/kjlm.2010.30.2.166.

Patil, R.; Patil, T.; Schenfeld, L.; Massoud, S. *Mycobacterium porcinum* peritonitis in a patient on continuous ambulatory peritoneal dialysis. *J. Gen. Intern. Med.* **2011**, *26*, 346–348. https://doi.org/10.1007/s11606-010-1571-y.

Chan, W.W.; Murray, M.C.; Tang, P.; Romney, M.G. *Mycobacterium heckeshornense* peritonitis in a peritoneal dialysis patient: A case report and review of the literature. *Clin. Microbiol. Infect.* **2011**, *17*, 1262–1264. https://doi.org/10.1111/j.1469-0691.2010.03449.x.

Jiang, S.H.; Senanayake, S.; Talaulikar, G.S. Peritoneal dialysis-related peritonitis due to *Mycobacterium smegmatis*. *Perit. Dial. Int.* **2011**, *31*, 215–216.

Renaud, C.J.; Subramanian, S.; Tambyah, P.A.; Lee, E.J. The clinical course of rapidly growing nontuberculous mycobacterial peritoneal dialysis infections in Asians: A case series and literature review. *Nephrology* **2011**, *16*, 174–179. https://doi.org/10.1111/j.1440-1797.2010.01370.x.

Simbli, M.A.; Niaz, F.A.; Al-Wakeel, J.S. Encapsulating Peritoneal Sclerosis in a Peritoneal Dialysis Patient Presenting with Complicated *Mycobacterium fortuitum* peritonitis. *Saudi J. Kidney Dis. Transpl.* **2012**, *23*, 635–641.

Siddiqi, N.; Sheikh, I. Peritonitis Caused by *Mycobacterium abscesses* in Patients on Continuous Ambulatory Peritoneal Dialysis. *Saudi J. Kidney Dis. Transpl.* **2012**, *23*, 321–324.

Jiang, S.H.; Roberts, D.M.; Dawson, A.H.; Jardine, M. *Mycobacterium fortuitum* as a cause of peritoneal dialysis-associated peritonitis: Case report and review of the literature. *BMC Nephrol.* **2012**, *13*, 35. https://doi.org/10.1186/1471-2369-13-35.

Jo, A.; Ishibashi, Y.; Hirohama, D.; Takara, Y.; Kume, H.; Fujita, T. Early Surgical Intervention May Prevent Peritonitis in Cases with Tenckhoff Catheter Infection by Nontuberculous *Mycobacterium*. *Perit. Dial. Int. J. Int. Soc. Perit. Dial.* **2012**, *32*, 227–229. https://doi.org/10.3747/pdi.2011.00080.

Lo, M.-W.; Mak, S.-K.; Wong, Y.-Y.; Lo, K.-C.; Chan, S.-F.; Tong, G.M.; Lo, K.-Y.; Wong, P.-N.; Tse, C.W.; Kam, K.-M.; et al. Atypical mycobacterial exit-site infection and peritonitis in peritoneal dialysis patients on prophylactic exit-site gentamicin cream. *Perit. Dial. Int. J. Int. Soc. Perit. Dial.* **2013**, *33*, 267–272. https://doi.org/10.3747/pdi.2011.00184.

Sangwan, J.; LathwaL, S; KuMar, S.; JuyaL; D. *Mycobacterium fortuitum* Peritonitis in a Patient on Continuous Ambulatory Peritoneal Dialysis (CAPD): A Case Report. *J. Clin. Diagn. Res*. **2013**, *7*, 2950–2951.

Miyasato, Y.; Adachi, M.; Fujie, Y.; Nakashima, Y.; Yamasaki, T.; Watanabe, M.; Sakanashi, A.; Tasaki, H.; Mizumoto, T.; Onoue, T.; et al. Severe Mycobacterium fortuitum infection due to inappropriate exit-site care using mountain spring water in a patient on continuous ambulatory peritoneal dialysis (CAPD). *Nihon Toseki Igakkai Zasshi* **2013**, *46*, 937–942. https://doi.org/10.4009/jsdt.46.937.

Ranganathan, D.; Fassett, R.; John, G.T. *Mycobacterium fortuitum* Peritonitis in a Patient Receiving Continuous Ambulatory Peritoneal Dialysis. *Saudi J. Kidney Dis. Transpl.* **2013**, *24*, 1003–1004,. https://doi.org/10.4103/1319-2442.118073.

Jiang, S.H.; Roberts, D.M.; Clayton, P.A.; Jardine, M. Non-tuberculous mycobacterial PD peritonitis in Australia. *Int. Urol. Nephrol.* **2013**, *45*, 1423–1428. https://doi.org/10.1007/s11255-012-0328-4.

Miyashita, E.; Yoshida, H.; Mori, D.; Nakagawa, N.; Miyamura, T.; Ohta, H.; Seki, M.; Tomono, K.; Hashii, Y.; Ozono, K. *Mycobacterium avium* complex-associated peritonitis with CAPD after unrelated bone marrow transplantation. *Pediatr. Int.* **2014**, *56*, e96–e98. https://doi.org/10.1111/ped.12463.

Kunin, M.; Knecht, A.; Holtzman, E.J. *Mycobacterium chelonae* peritonitis in peritoneal dialysis. Literature review. *Eur. J. Clin. Microbiol. Infect. Dis.* **2014**, *33*, 1267–1271. https://doi.org/10.1007/s10096-014-2079-x.

Hamade, A.; Pozdzik, A.; Denis, O.; Tooulou, M.; Keyzer, C.; Jacobs, F.; Khabbout, J.; Nortier, J.L. *Mycobacterium fortuitum* and Polymicrobial Peritoneal Dialysis-Related Peritonitis: A Case Report and Review of the Literature. *Case Rep. Nephrol.* **2014**, *2014*, 323757. https://doi.org/10.1155/2014/323757.

Zewinger, S.; Meier, C.-M.; Fliser, D.; Klingele, M. *Mycobacterium fortuitum* peritonitis in peritoneal dialysis and its effects on the peritoneum. *Clin. Nephrol.* **2014**, *82*, 341–346. https://doi.org/10.5414/cn107704.

Yamada, T.; Ushijima, K.; Uemura, O. A hospital-acquired outbreak of catheter-related nontuberculous mycobacterial infection in children on peritoneal dialysis. *CEN Case Rep.* **2015**, *4*, 43–47. https://doi.org/10.1007/s13730-014-0137-y.

Yang, T.-K.; Lee, J.-J.; Lu, P.-L.; Kuo, H.-T.; Kuo, M.-C.; Chen, H.-C. Peritoneal Dialysis-Associated Peritonitis Caused by *Mycobacterium abscessus*. *Perit. Dial. Int. J. Int. Soc. Perit. Dial.* **2015**, *35*, 369–371. https://doi.org/10.3747/pdi.2014.00012.

Inagaki, K.; Mizutani, M.; Nagahara, Y.; Asano, M.; Masamoto, D.; Sawada, O.; Aono, A.; Chikamatsu, K.; Mitarai, S. Successful Treatment of Peritoneal Dialysis-related Peritonitis due to *Mycobacterium iranicum*. *Intern. Med.* **2016**, *55*, 1929–1931. https://doi.org/10.2169/internalmedicine.55.5219.

Choi, H.-S.; Bae, E.-H.; Ma, S.-K.; Kim, S.-W. Peritoneal Dialysis-Related Peritonitis Caused by *Microbacterium paraoxydans*. *Jpn. J. Infect. Dis.* **2017**, *70*, 195–196. https://doi.org/10.7883/yoken.jjid.2016.004.

Fujikura, H.; Kasahara, K.; Ogawa, Y.; Hirai, N.; Yoshii, S.; Yoshihara, S.; Ogawa, T.; Yonekawa, S.; Imakita, N.; Nishioka, Y.; et al. *Mycobacterium wolinskyi* Peritonitis after Peritoneal Catheter Embedment Surgery. *Intern. Med.* **2017**, *56*, 3097–3101. https://doi.org/10.2169/internalmedicine.8871-17.

Chan, G.C.W.; Mok, M.M.Y.; Hung, D.L.L.; Chan, J.F.W.; Kwan, L.P.Y.; Ma, M.K.M.; Yap, D.Y.H.; Tang, S.C.W. *Mycobacterium chlorophenolicum*: An uncommon cause of peritonitis in a peritoneal dialysis patient. *Nephrology* **2017**, *22*, 498–499. https://doi.org/10.1111/nep.12820.

Mooren, V.H.; Bleeker, M.W.; van Ingen, J.; Hermans, M.H.; Wever, P.C. Disseminated Mycobacterium abscessus infection in a peritoneal dialysis patient. *IDCases* **2017**, *9*, 6–7.

Chin, H.H.; Chin, Y.H.; Yap, Y.L.; Chang, A.; Chua, H.H. Case Report: Two Cases of *Mycobacterium abscessus* Peritonitis in Patients on Continuous Ambulatory Peritoneal Dialysis (CAPD). *J. Nephrol. Res.* **2018**, *4*, 135–138. https://doi.org/10.17554/j.issn.2410-0579.2018.04.47.

Nakamoto, H. The Role of Non-Tuberculous Mycobacteria in Peritoneal Dialysis-Related Infections: A Literature Review. *Recent Adv. Dial. Ther. Jpn.* **2018**, *196*, 155–161.

Yoshimura, R.; Kawanishi, M.; Fujii, S.; Yamauchi, A.; Takase, K.; Yoshikane, K.; Egawa, M.; Shiina, H.; Ito, T. Peritoneal dialysis-associated infection caused by Mycobacterium abscessus: A case report. *BMC Nephrol.* **2018**, *19*, 341. https://doi.org/10.1186/s12882-018-1148-2.

Ono, E.; Uchino, E.; Mori, K.P.; Yokoi, H.; Toda, N.; Koga, K.; Kasahara, M.; Matsubara, T.; Yanagita, M. Peritonitis due to Mycobacterium abscessus in peritoneal dialysis patients: Case presentation and mini-review. *Ren. Replace. Ther.* **2018**, *4*, 52. https://doi.org/10.1186/s41100-018-0192-5.

Inoue, H.; Washida, N.; Morimoto, K.; Shinozuka, K.; Kasai, T.; Uchiyama, K.; Tokuyama, H.; Wakino, S.; Itoh, H. Non-Tuberculous Mycobacterial Infections Related to Peritoneal Dialysis. *Perit. Dial. Int. J. Int. Soc. Perit. Dial.* **2018**, *38*, 147–149. https://doi.org/10.3747/pdi.2017.00172.

Jheeta, A.S.; Rangaiah, J.; Clark, J.; Makanjuola, D.; Somalanka, S. *Mycobacterium abscessus*—An uncommon, but important cause of peritoneal dialysis-associated peritonitis—Case report and literature review. *BMC Nephrol.* **2020**, *21*, 491. https://doi.org/10.1186/s12882-020-02146-4.

Jung, J.H.; Ahn, S.-H. Peritoneal Dialysis Catheter-Related Infection due to *Mycobacterium abscessus* Confused with *Rhodococcus*. *J. Korean Med. Sci.* **2020**, *35*, e44. https://doi.org/10.3346/jkms.2020.35.e44.

Seki, M.; Kamioka, Y.; Takano, K.; Imai, H.; Shoji, M.; Hariu, M.; Oikawa, N.; Kabutoya, Y.; Watanabe, Y. *Mycobacterium abscessus* Associated Peritonitis with CAPD Successfully Treated Using a Linezolid and

Tedizolid Containing Regimen Suggested Immunomodulatory Effects. *Am. J. Case Rep.* **2020**, *21*, e924642-1–e924642-5, https://doi.org/10.12659/ajcr.924642.

Klomjit, N.; Chewcharat, A.; D'uScio, M.; Kattah, A.G. *Mycobacterium septicum* associated peritonitis: A case report. *Perit. Dial. Int. J. Int. Soc. Perit. Dial.* **2020**, *40*, 600–602. https://doi.org/10.1177/0896860820927150.

Imam, O.; Al-Zubaidi, K.; Janahi, M.; Imam, A.; Leghrouz, B.; Dobson, S.; Sundararaju, S.; Tsui, K.M.; Hasan, M.R.; Perez-Lopez, A. Peritoneal Dialysis–Associated Peritonitis Caused by *Mycobacterium abscessus* in Children—A Case Report. *Open Forum Infect. Dis.* **2021**, *8*, ofaa579. https://doi.org/10.1093/ofid/ofaa579.

Hamada, S.; Takata, T.; Kitaura, T.; Teraoka, C.; Aono, A.; Taniguchi, S.; Mae, Y.; Isomoto, H.; Chikumi, H.; Mitarai, S. Peritoneal dialysis-associated peritonitis caused by *Mycobacteroides massiliense*: The first case and review of the literature. *BMC Nephrol.* **2021**, *22*, 90. https://doi.org/10.1186/s12882-021-02297-y.

Lu, J.; Jiang, Z.; Wang, L.; Mou, S.; Yan, H. Mycobacteria avium-related peritonitis in a patient undergoing peritoneal dialysis: Case report and review of the literature. *BMC Nephrol.* **2021**, *22*, 345. https://doi.org/10.1186/s12882-021-02544-2.

Pinapala, A.; Koh, L.J.; Ng, K.-H.; Tambyah, P.A.; Yap, H.-K. Clofazimine in *Mycobacterium abscessus* peritonitis: A pediatric case report. *Perit. Dial. Int. J. Int. Soc. Perit. Dial.* **2021**, *41*, 104–109. https://doi.org/10.1177/0896860820909702.

Yokota, S.; Nishi, K.; Ishiwa, S.; Uda, K.; Shoji, K.; Kamei, K. Mycobacterium avium complex peritonitis in a pediatric patient on peritoneal dialysis: A case report. *Medicine* **2021**, *100*, e26321.

Chang, Z.Y.; Tok, P.L.; Teo, B.W.; Hong, W.; Tambyah, P.A.; Lee, M.B.-H. Non-tuberculous mycobacteria infections in peritoneal dialysis: Lessons from a 16-year single-centre experience. *Ann. Acad. Med. Singap.* **2021**, *50*, 724–728. https://doi.org/10.47102/annals-acadmedsg.2021228.

Masuda, K.; Yamasaki, Y. A successfully treated case of PD–related peritonitis caused by Mycobacterium wolinskyi. *Nihon Toseki Igakkai Zasshi* **2022**, *55*, 249–253. https://doi.org/10.4009/jsdt.55.249.

Haubrich, K.; Mammen, C.; Sekirov, I.; Mitchell, H. *Mycobacterium fortuitum* peritoneal dialysis-related peritonitis in a child: A case report and review of the literature. *Off. J. Assoc. Med. Microbiol. Infect. Dis. Can.* **2022**, *7*, 125–130. https://doi.org/10.3138/jammi-2021-0029.

Hayat, A.; Sakhrani, B.; Rubin, M. *Mycobacterium chelonae*-related peritoneal dialysis peritonitis: A case report and its potential complications. *Int. Urol. Nephrol.* **2022**, *54*, 1769–1771. https://doi.org/10.1007/s11255-021-03039-7.

Nakano, S.; Yamamura-Miyazaki, N.; Michigami, T.; Yazawa, K.; Yanagihara, I.; Yamamoto, K. A case of a preschool child with a successful kidney transplant following the long-term administration of antibiotics to treat peritoneal dialysis-related ESI/peritonitis by *Mycobacterium abscessus*. *CEN Case Rep.* **2022**, *11*, 408–411. https://doi.org/10.1007/s13730-022-00689-z.

Rouhani, S.; Adunuri, N. Refractory Peritonitis and Small Bowel Ileus: A Case of Encapsulating Peritoneal Sclerosis Secondary to *Mycobacterium abscessus* Peritonitis. *Eur. J. Case Rep. Intern. Med.* **2022**, *9*, 003173. https://doi.org/10.12890/2022_003173.

Ueda, Y.; Okamoto, T.; Sato, Y.; Hayashi, A.; Takahashi, T.; Kamada, K.; Honda, S.; Hotta, K. Kidney transplantation after peritoneal dialysis-associated peritonitis and abdominal abscesses caused by *Mycobacterium massiliense*: Lesson for the clinical nephrologist. *J. Nephrol.* **2022**, *35*, 1907–1910. https://doi.org/10.1007/s40620-021-01244-2.

Chen, X.; Zhu, J.; Liu, Z.; Ye, J.; Yang, L.; Zhang, Z. Mixed infection of three nontuberculous mycobacteria species identified by metagenomic next-generation sequencing in a patient with peritoneal dialysis-associated peritonitis: A rare case report and literature review. *BMC Nephrol.* **2023**, *24*, 95. https://doi.org/10.1186/s12882-023-03156-8.
